# Notes from a Lecture upon Spongia

**Published:** 1887-01

**Authors:** 

**Affiliations:** Hahnemann Medical College, Chicago


### NOTES FROM AN EXTEMPORANEOUS LECTURE ON SPONGIA.

PROFESSOR GEE, OF HAHNEMANN MEDICAL COLLEGE, CHICAGO.

This valuable remedy comes to us from Hahnemann. It is not one of his deep, long-acting "antipsorics," but has proved to be of great value even in its limited sphere. In a domestic way it had been used for the cure of goitre, but in large and repeated doses of the powder.

That it has been of great value in the cure of this trouble many homœopathicians will testify. Some have attributed this power to the slight amount of Iodine in the sponge.

No peculiar mental symptoms are deserving of special mention, but it has produced a dull, right-sided headache coming from cold air into a warm room. The headache is at times "congestive," with pressing, beating in forehead, red face. All the headaches are relieved by lying on the back horizontally.

Mouth and throat full of vesicles, with burning and stinging pains. You will remember that similar conditions exist when Nat. mur., Staph., and some other remedies are needed. When Spongia is the remedy the saliva is diminished (except, clinically, in whooping-cough), and the burning, stinging pains are present. When Staph. is preferred there is the constant accumulation of saliva, which necessitates frequent swallowing. The Nat. mur. vesicles are very sensitive to touch, even of food and liquids. The distress, too, is more of a smarting character.

Spongia is full of stuffed, cramped-like sensations. The parts thus disturbed are sensitive to touch, and soreness and stitches are likely to accompany.

Fullness of head as if skull would burst; congestion of blood to ears; nose stuffed up.

The burning and stinging of the mouth is also present in the throat, and a peculiarity of the sore throat is that it is worse after eating sweet things. A similar condition is present under Sang., especially when the burning is thus aggravated.

On the outside the throat is greatly swollen, "even with the chin," as the text has it. When called for, the throat is likely to be sensitive to touch; and although the throat and general symptoms of the patient are better when lying down, he frequently wakes frightened because of suffocating spells. The stomach symptoms conform to the features of the remedy in being aggravated from touch of clothing; better lying on back. There is also a drawing, as if growing together, of pit of stomach even to throat, obstructing his breathing; stitches in stomach. This is the cramp-like pain in different language. Later this cramp relaxes and leaves the parts flaccid, and then we have the very peculiar sensation as if the stomach was standing open. (When the os uteri feels as if open—Lach.; when it really does stand wide open—Kre.)

The abdominal muscles share in this drawing of the stomach, making inspiration very difficult.

Spongia has proved to be of value in troubles affecting the genital organs. Testicles swollen and hard, as in orchitis; sensitive to touch, with stitches. A peculiar squeezing, screwing pain is present, as under Coloc. and Staph.

We now come to a valuable sphere for this remedy, and one in which its greatest laurels have been won, viz.: disease of the larynx and chest.

The same stuffed sensation is in the larynx; obstruction as from a plug; it produces real inflammation of the respiratory track. In the beginning is dryness, sensitiveness to touch, voice cracked, gives out, cough, hoarse, talking hurts, and even turning the neck causes pain. The stiffness, with the soreness and sensitiveness to touch which runs through the remedy, is here present. The sensation is as if grasped about the throat, causing the fright and suffocation on waking. The same grasping is present under Acon. Is it surprising that this remedy should be so valuable in croup?

You will observe that from the character of the "plugging" of the larynx the difficult breathing is on inspiration. This is a keynote: Wheezing, anxious, labored inspiration, even involving the abdominal muscles. This is an objective symptom, and you need not mistake it when called to a case of croup. We now find a marked exception to the general action of the remedy. The chest troubles are worse lying down; the breathing is worse lying down, but better bending forward. (You remember the Hepar sul. patient must sit up and bend backward.) The same stuffed, bursting feeling is present in the chest. The cramplike condition gives a stuffed feeling, as though he had to breathe through a dry sponge.

A short time ago a patient came slowly into the office, walked stooped, sat down carefully, leaned forward and kept very quiet, and seemed to be much exhausted. When he had rested a moment he said he had frequent attacks of asthma, but this was the worst yet experienced. The labored inspiration involving the abdominal muscles was quite marked. He answered that he had not slept for two nights and had been obliged to sit upright in his chair; that leaning backward or lying down or exertion "took his breath away." He received one dose on his tongue of Spongia$^{1m}$ (B. & T.), and within two minutes (I thought hardly time to dissolve or swallow the powder) he threw back his shoulders and, with an expression peculiarly his own, remarked that he did not know what I gave him, but "it went right to the spot" and "something has let up." He was able to walk out in a few minutes relieved and a happy man.

The cough is dry and barking and, like the sore throat, is worse from sweats, also from lying down with the head low, and from excitement. It is worse from cold and better from warm foods and drinks. It is true of Spongia, as with other remedies, that when one extreme is present, in the latter stage the opposite may be expected, even if not recorded. If a man takes whisky he is stimulated for a time, but the after effect is greater prostration than before taking it. So with a drug. If diarrhœa occurs first, constipation will follow, and vice versa.

Dry cough occurs first under Spongia, but it may be indicated in loose cough with profuse sputa. The croup cough at times sounds like sawing on a board, and the same sound is present between coughs, but it may be found useful in chronic coughs of a paroxysmal character when the mucus "torn loose" must be swallowed, as under Arn., Caust., and Sep., or is in a hard lump, as under Ox. ac. and Mag. carb.

This remedy has proved valuable for angina pectoris when the contracting cramplike pain is present with the suffocating spells, and worse with the head low. Think also of Rumex and Cactus grand. for many of these symptoms.

The cramplike pain with stiffness also extends into the muscles. We find it in the left side of the face—articulation of the jaw; in the muscles of the left shoulder; in the ball of the right thumb—in the muscles about a joint.

In sleep, the old position is resumed—wants the head low. (The desire to lie with the head low has led to Arn. in serious diseases.) Awakes frightened; starts, because he fears he will suffocate. (The Sul. patient wakes with a start and a scream. The Stram. is greatly frightened at what he sees first. The Lyc. patient is cross, cries and kicks on waking. The Lach. is generally worse after sleep—mentally and otherwise. His disease grows worse while he is asleep.)

Remember, these features characterize the remedy, and do not attempt to memorize each single symptom:

*Sensitive to touch.*

*Cramplike pains, with soreness, stiffness, and stitches*—3 " S's."

*Relief from lying down of all troubles except those of the chest.*

*Suffocative attacks.*

*Chest troubles—lying down, better leaning forward. Stuffed, obstructed sensation, with difficult inspiration.*

